# Mix-and-matching as a promoter recognition mechanism by ECF σ factors

**DOI:** 10.1186/s12862-016-0865-z

**Published:** 2017-02-07

**Authors:** Jelena Guzina, Marko Djordjevic

**Affiliations:** 1 Institute of Physiology and Biochemistry, Faculty of Biology, University of Belgrade, Studentski trg 16, 11000 Belgrade, Serbia; 2 Multidisciplinary PhD program in Biophysics, University of Belgrade, Belgrade, Serbia

**Keywords:** Bacterial promoters, Transcription initiation, σ^70^ family, Mix-and-matching, ECF σ factors

## Abstract

**Background:**

Transcription initiation is in bacteria exhibited by different σ factors, most of which fall within σ^70^ family. This family is diverse, ranging from the housekeeping Group I (RpoDs), to Group IV (ECF) σ factors, that transcribe smaller regulons under more stringent conditions. RpoDs employ a kinetic mix-and-match mechanism, where promoter elements complement each other binding strengths in achieving sufficient transcription activity. On the other hand, it is assumed that ECF σs, which are the most distant from the housekeeping σ factors, cannot exhibit mix-and-matching. However, mix-and-matching for ECF σ factors was not quantitatively checked before, and recent results show a much larger flexibility in the promoter recognition by the members of this group.

**Results:**

To this end, we quantitatively investigate mix-and-matching in two canonical ECF σ family members (σ^E^ and σ^W^), for which we use a biophysics based model of transcription initiation. For σ^E^, we perform a separate analysis for in-vitro *active* and in-vitro *inactive* promoters, which allows us investigating how mix-and-matching depends on the external factors that may control transcription activity in the in-vitro *inactive* set. We show that the promoter elements of canonical ECF σs significantly complement each other strengths, where such mix-and-matching is in the in-vitro *active* set even stronger compared to the correlations observed for the housekeeping σs. This complementation however significantly decreases for the in-vitro *inactive* set, which we propose is due to mix-and-matching with regulatory sequences outside of the canonical promoter elements. In line with this proposition, we show that a conserved spacer element, which appears in the in-vitro *inactive* promoter set, significantly increases the promoter element complementation. While RpoD promoter elements mix-and-match to achieve sufficient total transcription activity, for σ^E^ they complement each other to achieve sufficiently strong total binding affinity, which we relate to differences in physiological responses between the two groups of σ factors.

**Conclusion:**

Despite a common notion that smaller σ factor specificity leads to a larger mix-and-matching, we here obtain a larger promoter element complementation for σ^E^ compared to RpoDs. Finally, to explain this finding, we propose a simple model which relates the size of σ factor regulon with the extent of mix-and-matching, based on an assumption of a selection pressure on promoters that are near the non-specific binding boundary to remain functional.

**Electronic supplementary material:**

The online version of this article (doi:10.1186/s12862-016-0865-z) contains supplementary material, which is available to authorized users.

## Background

**Fig. 1:**
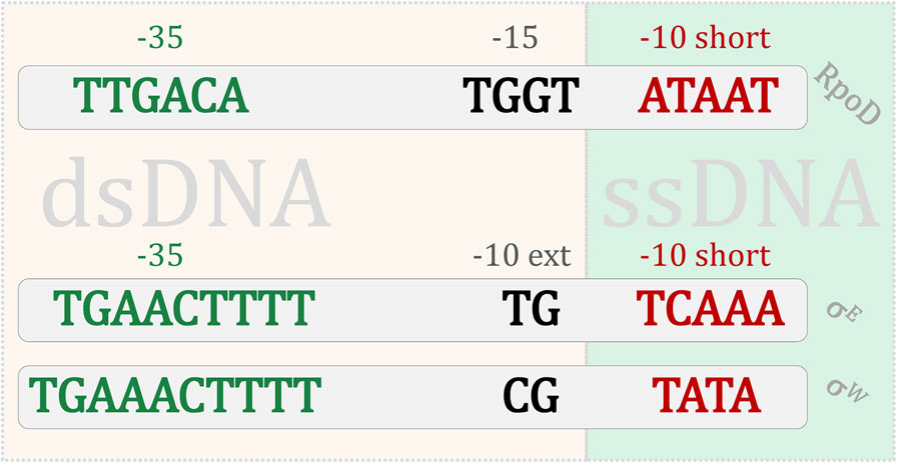
Promoter structure in σ^70^ family: The promoter organization for RpoD (*E. coli* RpoD in the upper part of the figure) and ECF group (*E. coli* σ^E^ and *B. subtilis* σ^W^ in the lower part of the figure) is shown; the promoter elements implicated in dsDNA interactions with σ factor are shown in *green/black*, and *shaded* with the *red rectangle*; the promoter elements implicated in ssDNA interactions with σ factor are shown in *red*, and *shaded* with the *green rectangle*

RNA polymerase holoenzyme (RNAP) is a major enzyme, in charge of transcription in prokaryotes, which consists of a core RNA polymerase in complex with a σ factor. The core RNA polymerase catalyzes the reaction of phosphodiester bond formation in a growing RNA chain, which is preceded by transcription initiation exhibited through σ factor interactions with DNA promoter elements [1]. Different σ factors govern the transcription under different conditions, and most of the known σ factors belong to the σ^70^ family. Promoters which are transcribed by this family share the same general structure [1, 2], whose hallmark are two canonical, −35 and −10, promoter elements. To initiate transcription, RNAP binds to double-stranded (dsDNA) promoter elements, and subsequently triggers the formation of a transcription bubble within −10 element. As a consequence, −35 and the upstream segment of −10 element (often called the extended −10 element, or −15 element), accomplish their σ factor-interactions in a double-stranded (dsDNA) form, while the downstream segment of −10 element (short −10 element) accomplishes its σ factor-interactions in a single-stranded (ssDNA) form (Fig. 1) [1].

σ^70^ family consists of 4 different subfamilies (Groups I to IV), where protein sequences between subfamilies are significantly different at the level of structural complexity despite the general similarity in their promoter recognition mechanisms. Group I (also named RpoD) σ factors are responsible for the majority of cellular transcription (i.e. transcribe the housekeeping genes), which makes them indispensable for functioning of the cell under normal conditions [1, 3]. Group II has a structure that closely resembles the Group I’s (i.e. has four analogous σ domains), however, the cell survival does not depend on the activity of the Group II members [1, 3]. Groups III (which has three domains σ_2_ - σ_4_) and IV (which has just two domains σ_2_ and σ_4_) [1], also known as alternative σ factors, are recruited by the cell under specific conditions (in response to either developmental or external signals), so that their regulons are much smaller compared to those of RpoDs.

Group IV σs, also named ECF (*E*xtra*C*ytoplasmic *F*unction), which are by far the most abundant alternative σ factors, are activated by the stimuli from the cell exterior to either help the cell cope with various stressors or supply specific nutrients. In line with this, it is considered that ECF members have to exhibit a fast response to the activating external signals, which is accomplished through the interactions of the ECF domains σ_2_ and σ_4_ with −10 and −35 promoter elements, respectively. Consistent with this notion, most of the ECF σ factors are autoregulated, so that the fast responsiveness is facilitated by the existence of a positive feedback loop.

Despite the structural and functional diversity within σ^70^ family, the mechanism of transcription initiation was well studied only for RpoDs (Group I) [1, 2, 4], which have been found to exhibit mix-and-match mode of action [5]. The initial observation has been that different promoter elements, which interact with σ factor in dsDNA form, may complement each other for achieving a sufficient level of the binding strength to dsDNA, thus providing a sufficiently efficient first kinetic step in transcription initiation. A finding that in RpoDs the extended −10 element can compensate for an absence of −35 element [1, 5] is altogether the best known example and extreme qualitative signature of the mix-and-matching mechanism.

Consequently, the initial mix-and-match proposal has been that the strengths of the promoter elements – that interact with σ factor in dsDNA form – complement each other [5]. For example, the promoters with the extended −10 element have been found to contain more mismatches in their −35 elements compared to the promoters that lack this element [6]. On the other hand, a systematic quantitative analysis that we subsequently performed has pointed to a different picture, where both ssDNA and dsDNA-interacting promoter elements complement each other strengths, to achieve a sufficiently high level of transcription activity [7]. This finding, also supported by the available biochemical measurements, opposes the classical viewpoint that the promoter elements mix-and-match so as to achieve sufficiently strong RNAP binding [8, 9]. While mix-and-matching has been well established for RpoDs, it is not self-evident that it should occur, particularly since the promoter strengths can differ for almost two orders of magnitude [10]. For example, one can imagine promoter elements working together to enforce high transcription activity that may be necessary for some promoters. Therefore, a question that remains to be understood is how mix-and-matching relates to possible other constraints on promoter sequences.

In distinction to RpoDs, mix-and-matching is considered to be absent in Groups III and IV (ECF) σs [1, 11]. This viewpoint, however, contradicts the intuitive notion that there should be a selection pressure for keeping promoter functionality, i.e. to preserve transcription links in a sigmulon (an equivalent to regulon for σ factors). More precisely, mutations in one promoter element, which decrease its interaction energy with σ factor, may be compensated by mutations in another promoter element with the opposite effect on its σ factor interaction energy, thus preserving a minimal value of the relevant kinetic parameter. Moreover, all factors in σ^70^ family initiate transcription in biophysically equivalent manner, where binding to dsDNA of −35 and extended −10 (−15) element is followed by opening of the two DNA strands in short −10 element [12, 13]; consequently, it may be expected that there should be a common kinetic mechanism of promoter recognition, such as mix-and-matching. Moreover, mix-and-matching may exploit not only interactions of the promoter elements with σ factors, but also external promoter signatures, such as those related with the interactions with enzymes of core RNA polymerase (e.g. with αCTD or β and β^’^subunits) [14–18], which may additionally enhance mix-and-matching.

On the other hand, mix-and-matching can mechanistically be implemented with significant differences for various σ^70^ subfamilies. Namely, σ^70^ factors differ greatly in terms of their structure and nature of the executed physiological response, thus making plausible that different kinetic parameters define functional promoter in different σ^70^ groups; this could be accomplished through mix-and-matching of different combinations of bacterial promoter elements. Consequently, investigating the correlations between the relevant promoter element strengths may also provide important information about the mechanism of transcription, such as which kinetic parameters (e.g. a binding affinity or transcription activity) define a functional promoter for a given σ^70^ group.

To assess the issue above, i.e. if mix-and-matching is present in σ^70^ family outside of RpoDs, we here concentrate on investigating this mechanism in ECF σ subfamily, which is plausible due to the following:

i) ECF σ factors are both structurally and functionally the most divergent from Group I σs within σ^70^ family [19]. Consequently, establishing mix-and-matching within ECF σ factor group might suggest its presence in the entire σ^70^ family, as Groups II and III are closer to Group I (RpoDs) than ECFs.
ii) We have recently done a detailed analysis of the protein and DNA interaction motifs which are involved in the promoter recognition by ECF σ factors [20]. Contrary to the previous considerations that ECF σ factors require a rigid promoter structure with highly conserved elements, this analysis revealed a substantial flexibility in ECF σ - promoter interactions. In particular, we showed that ECF σ promoters (in particular those found in bacteriophages) can contain an extended −10 element, which interacts with an ECF σ factor segment, located just C-terminal of domain σ_2_. Interestingly, in canonical ECF σ factors (σ^E^ and σ^W^) a similar motif was also found C-terminal of domain σ_2_, positioned exactly to interact with a conserved element in the promoter spacer sequence – whereby this conserved spacer element was previously not recognized to be involved in interactions with ECF σ factors. The observed larger flexibility suggests that ECF σs might also employ mix-and-matching during promoter recognition. In particular, the appearance of the extended −10 element, which is in bacteriophages accompanied by a complete absence of a recognizable −35 element, is a classical (qualitative) signature of mix-and-matching [5].
iii) While transcription initiation mechanisms for alternative σ factors are generally poorly studied, for σ^E^ (a canonical ECF σ member) there is a relatively large promoter set, whose in-vitro transcription activity was assessed under the same conditions (i.e. within a single experiment) [21]. This allows dividing σ^E^ promoter set to those that are *active* and *inactive* in-vitro, where such separation will allow us to investigate what kinetic parameters determine functional promoters within each subset. Moreover, another canonical ECF σ family member (σ^W^) has a number of experimentally characterized promoters, which makes it a suitable candidate for our analysis.

Consequently, we will here systematically investigate mix-and-matching for ECF σ factors, by concentrating on two canonical subfamily members, σ^E^ and σ^W^. We will also perform a wider analysis of mix-and-matching in RpoDs, since comparison of these results with the ones obtained for ECF σs will allow analyzing how mechanistic differences in the two σ^70^ groups influence the observed differences in mix-and-matching that we will infer. More precisely, the observed mechanistic differences (i.e. which parameter is relevant for promoter kinetics) will be discussed in the context of distinct structural and functional constraints, that exist for different σ^70^ groups; on the other side, differences in magnitude of the observed mix-and-matching effect will be discussed in the context of a model that we propose, which relates the extent of mix-and-matching with the relevant sigmulon size.

## Results

### Quantitative analysis of mix-and-matching

**Fig. 2:**
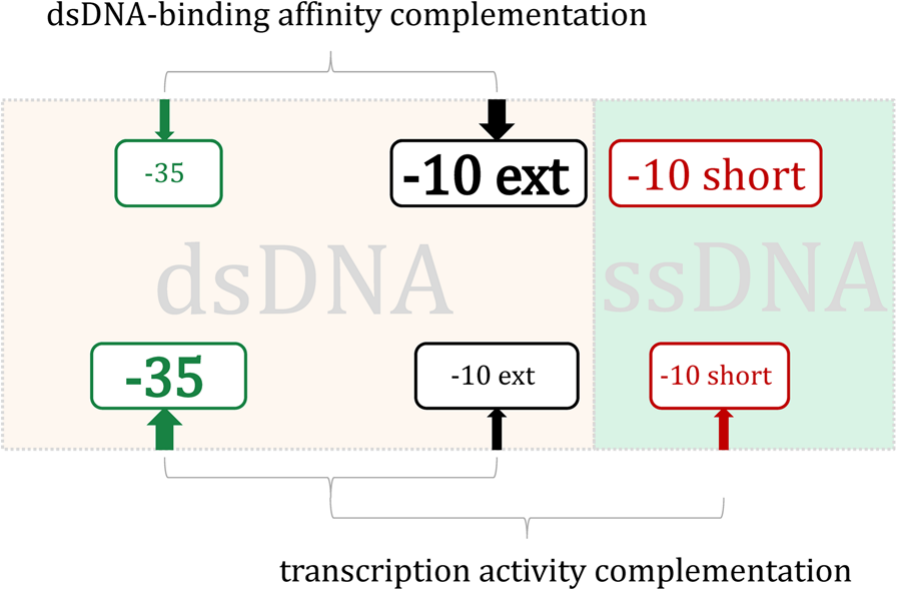
Different modes of the mix-and-matching mechanism: The promoter elements participating in mix-and-matching are divided to dsDNA (*shaded in red*) and ssDNA-interacting (*shaded in green*); In the upper part of the figure the complementation between the strengths of the dsDNA-interacting elements for achieving sufficient level of dsDNA-binding affinity is shown; in the lower part of the figure the complementation between the strengths of the dsDNA and ssDNA-interacting elements for achieving sufficient level of transcription activity is shown; the strength of the promoter element which is indicated by the size of the corresponding font and *arrow*

To assess the mix-and-matching mechanism in ECF subfamily we quantitatively analyze the canonical ECF members, through a biophysics-based model of transcription initiation (see Methods). The analysis will be done analogously to RpoD group (for *E. coli* RpoD factor), by correlating the weight matrix scores of the relevant (dsDNA and ssDNA-interacting) promoter elements [7]. The weight matrix scores provide a measure of the promoter element strengths, i.e. of the corresponding DNA binding energies, under the widely used unsaturated approximation [13, 22]. Consequently, strengths of the promoter elements that interact with σ factor in dsDNA form contribute additively to the log of the binding affinity, as illustrated in Fig. 2 [13, 23]. Similarly, adding also strengths of the promoter elements that interact with σ factor in ssDNA form – that is, including both ssDNA and dsDNA-interacting promoter elements – gives an estimate of the log total promoter strength, which corresponds to the promoter transcription activity under the unsaturated approximation (see refs. [10, 13] and also summarized in Methods).

The analysis will be systematically done in the following way: the promoter elements are divided in those that interact with σ factor in dsDNA form (−35 and extended −10 elements), and in ssDNA form (short −10 element); note that the spacer length (through spacer weights) also contributes to both the total promoter strength and to dsDNA binding strength (proportional to the log binding affinity). Finally, note that when comparing the strength of a given promoter element with the relevant kinetic parameter (dsDNA binding affinity or transcription activity), the element strength is excluded from the parameter, to avoid correlating with itself.

**Fig. 3:**
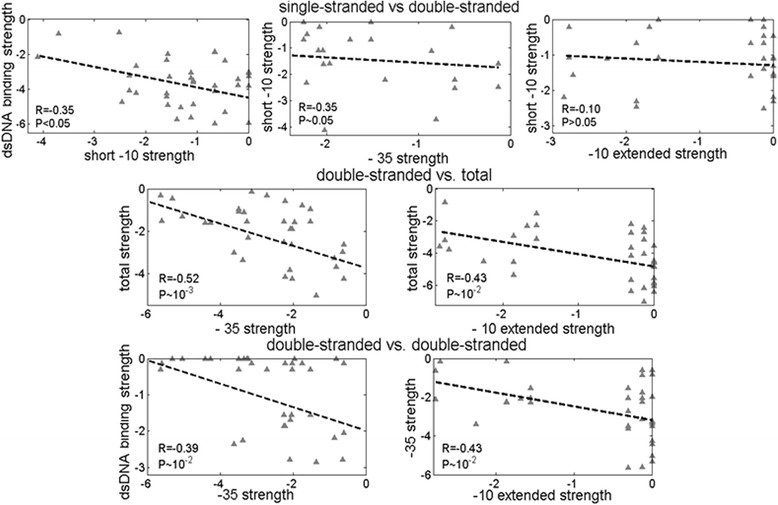
σ^E^ in-vitro *active* promoter mix-and-matching: Correlations between the promoter elements, for the in-vitro *active* promoters, are shown in the figure panels. The promoter elements whose strengths are correlated are, for each panel, indicated on the axes. The correlation constants and *P* values are also indicated for each panel. Strengths of individual promoter elements are estimated by the corresponding weight matrix scores (see Methods). Note that the total promoter strength, and dsDNA binding strength, correspond, respectively, to the sum of all the element strengths (Eq. 1.7), and the sum of the element strengths involved in dsDNA binding (Eq. 1.6) – with the element involved in the correlation excluded from the sum. The first row correspond to the correlation of the dsDNA and ssDNA interacting elements; the second row to the correlations between dsDNA interacting elements and the total promoter strength; the third row to the mutual correlations of dsDNA interacting elements

Correlating ssDNA and dsDNA element strengths with each other, or with dsDNA binding strength and the total promoter strength, allows directly assessing mix-and-matching between the elements; i.e. complementation of one weaker element by another stronger element leads to negative correlations between the relevant strengths (assessed by the weight matrix scores). Furthermore, as the element strengths are complemented to achieve a sufficient level of the relevant kinetic parameter, we also aim identifying this parameter. Consequently, for allowing easier interpretation, the results in the Fig. 3 below are organized in the following way: the correlations of single-stranded with double-stranded element strengths (the first row in the figure panel), which indicate the complementation towards achieving sufficiently high transcription activity; correlations of double-stranded element strengths with total promoter strength (the second row in the figure panel) and mutual correlations of the double-stranded element strengths (the third row in the panel), which indicate the complementation towards achieving sufficiently high binding affinity to dsDNA.

### Correlating σ^E^ promoter elements strengths

We start by examining σ^E^ promoters that are *active* in-vitro, where the results are shown in Fig. 3. Note that these promoters have the transcription activity level above the established threshold [21], so that their activity is determined solely by the intrinsic properties of their basal elements, which we consider in our initial analysis. Statistically significant correlations between almost all the elements/parameters in Fig. 3 can be immediately noticed; in fact, these correlations are noticeably stronger compared to those found in *E. coli* RpoD [7], where mix-and-matching is well established. For example, one can observe significant negative correlations between the single-stranded and the double-stranded promoter elements (the first row in the panel), and between the double stranded promoter elements and the total promoter strength (the second row), which clearly indicates complementation towards achieving a sufficient level of transcription activity, as also observed in *E. coli* RpoD [7]. Note that by significant correlations we here consider those that are statistically significant, i.e. with *P* values at 5% confidence level or lower. Despite the statistical significance, a notable scatter may appear in the correlation plots: this is both due to a limited size of the dataset, and also likely due to inherent properties of mix-and-matching, as proposed by the model that we present in Discussion.

Furthermore, as also stated in the previous subsection, note that the total promoter strength and dsDNA binding strength in Fig. 3 involve strengths of several individual promoter elements. For example, one can observe a higher correlation between −35 strength and the total promoter strength (the second row) than between −35 element strength and short −10 element (the first row). This is a consequence of the fact that the total promoter strength involves both the extended −10 element strength and the spacer weight, in addition to the short −10 element strength (note that −35 element strength is excluded from the total promoter strength to prevent self-correlations). That is, the higher correlation with the total promoter strength is due to significant correlations of −35 element with dsDNA binding elements, as can be observed in the third row of Fig. 3.

Finally, in the third row of Fig. 3 one can notice a subpopulation of promoters with high extended −10 element strength and dsDNA binding strength (note that the high scores correspond to values close to zero), which shows a largely unrelated strengths of −35 and extended −10 elements – this subpopulation contributes to the visual appearance of the scatter in the two plots. From this one may conclude that a strong extended −10 element makes the −35 element strength much less important in terms of mix-and-matching [5]. This observation is in fact analogous to the well-known result in the housekeeping σ factors that the presence of a strong extended −10 element can compensate for an absence of −35 element, which is interpreted by the promoter being able to achieve a sufficient value of the binding affinity with just the strong extended −10 element, regardless of the −35 element strength.

As the main difference with respect to RpoDs, we previously found that strong negative correlations between dsDNA elements in RpoDs are absent. However, as can be seen in the third row of Fig. 3, for σ^E^ we now observe significant negative correlations between the double stranded promoter elements. Consequently, while for ECF σ factors we observe generally stronger complementation of the promoter elements than in RpoDs, the main difference is strong mix-and-matching in achieving sufficient binding affinity that appears in ECF σs.

Furthermore, mix-and-matching that we find for *E. coli* σ^E^ promoters, can also be observed for *B. subtilis* σ^W^ promoters, tough the analysis is here complicated by a smaller promoter dataset, and a notably stronger conservation compared to σ^E^ [24]: In particular, −10 element is much more conserved in σ^W^ than in σ^E^, with no more than two mismatches from the consensus; similarly, the extended −10 element is almost completely conserved, with one mismatch appearing in only few of the promoters. Therefore, we construct weight matrices for −35 elements (which display sufficient variability), while for −10 element we divide σ^W^ promoter set in three groups: those having zero, one and two mismatches; we then estimated (average) -35 element strengths for each of the groups. We obtain that weaker −35 element strengths are associated with zero mismatches compared to one mismatch, which, in turn, show weaker strengths compared to two mismatches (Additional file 1). Consequently, we obtain that the larger number of mismatches in −10 element (i.e. a weaker −10 element), leads to stronger −35 element strength, which is the tendency consistent with mix-and-matching.

**Fig. 4:**
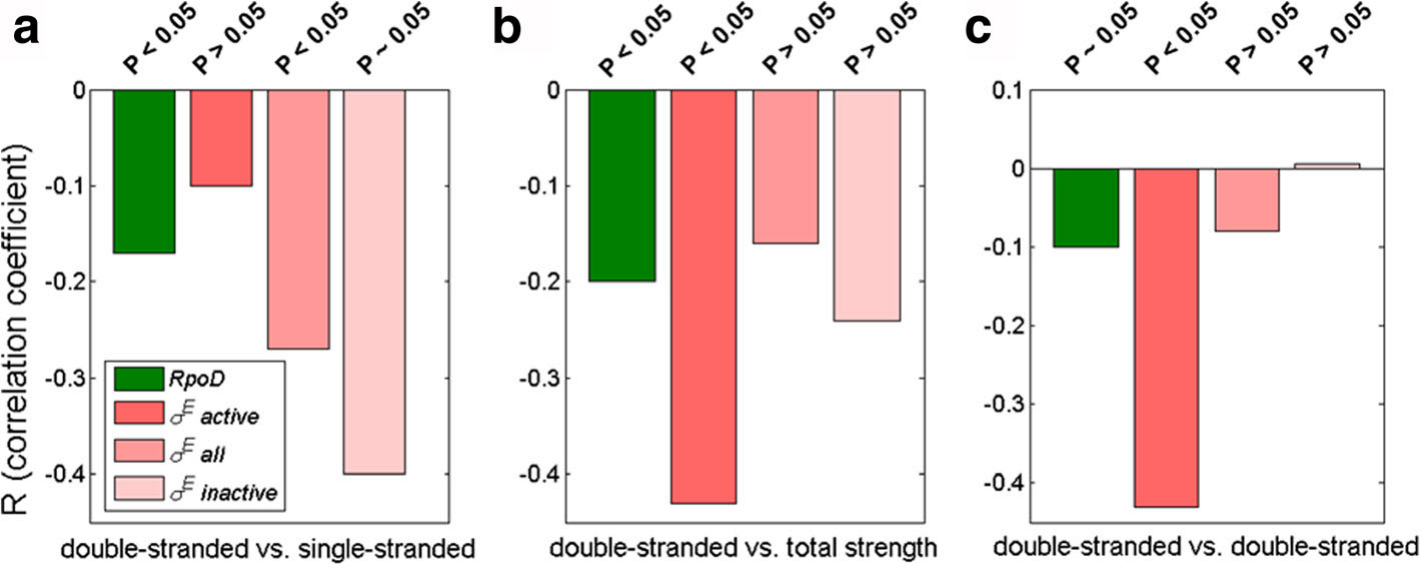
From in-vitro *active* to in-vitro *inactive* promoters (comparison with RpoD). Complementation between: **a** double-stranded (represented by extended −10) and single-stranded (represented by short −10) **b** double-stranded (extended −10) and total strength, **c** double-stranded (−10 extended) and double-stranded (−35) promoter elements is shown for in vitro *active*, *all*, and in vitro *inactive* σ^E^ promoters. The relevant correlations (indicated on the *y*-axis) are compared with the corresponding values for RpoD (where −15 element is used). The information on the relevant promoter datasets is indicated in the figure legend

Next, to gain an understanding of how mix-and-matching is affected by increasing heterogeneity in the promoter dataset, we go back to σ^E^ promoters, and include in the analysis those promoters that are *inactive* under in-vitro conditions. Therefore, we further analyze complementation between the promoter elements in two additional datasets: *i*) *all* σ^E^ promoters which include both in-vitro *active* and in-vitro *inactive* promoters *ii*) in-vitro *inactive* promoters. In Fig. 4, we compare the correlations in these two new promoter sets with those previously observed in in-vitro *active* set – i.e. we assess how the correlations change, as we move from in-vitro *active*, to *all*, to in-vitro *inactive* promoters. The comparison is done for complementation of double-stranded vs. single stranded promoter elements (panel A), double-stranded vs. total promoter strength (panel B), and double-stranded vs. double stranded promoter elements (panel C). The representative correlations for these three σ^E^ promoter sets are then compared with the representative correlations for RpoD promoters (the leftmost bars in the panels).

In RpoD sequences, the strongest observed complementation of the promoter elements was towards achieving sufficient total transcription activity [7]. As can be seen in Fig. 4a (compare the first and the second bar), a smaller correlation is observed for the in-vitro *active* σ^E^ sequences (−0.1 for σ^E^ compared to −0.17 for RpoD). These correlations however increase (from −0.1 to −0.4) as one moves towards the in-vitro *inactive* sequences (compare bars 3–4). This increase is likely a consequence of the fact that in-vitro *inactive* sequences are under a pressure to increase their inherently low transcription activity through mix-and-matching.

A reverse trend is observed for the complementation of dsDNA binding elements, as can be seen in Fig. 4c (the rightmost panel). There, we see significantly stronger negative correlation between the strengths of dsDNA binding elements for σ^E^ in-vitro *active* promoters compared to RpoD promoters. This then underscores the main difference between the transcription kinetics for σ^E^ and RpoD. While in RpoD it is the total transcription activity that defines a functional promoter, in σ^E^ the promoter elements mainly complement to achieve a sufficiently high binding affinity to dsDNA.

Moreover, the correlations between dsDNA binding elements decrease as one moves from the in-vitro *active* to in-vitro *inactive* sequences (compare bars 2–4 in Fig. 4c), which further confirms that σ^E^ promoter activity is related to binding affinity to dsDNA. In particular, the correlations between dsDNA binding elements decrease from significant negative values (−0.43) observed for the in-vitro *active* sequences, to the absence of correlations observed for the in-vitro *inactive* sequences. This pattern of correlations observed for dsDNA binding elements, induces a similar trend for the correlations between dsDNA binding elements and total promoter strength, as can be observed in Fig. 4b (the central panel).

This significant decrease of correlations observed in Fig. 4b and c when moving from in-vitro *active* to in-vitro *inactive* σ^E^ promoters may be a consequence of the fact that the activity of the in-vitro weak promoters likely depends on external regulatory elements. These external elements may become involved in mix-and-matching that is not accounted for by the correlations between the canonical promoter elements. This external contribution to dsDNA binding affinity (and to mix-and-matching) might be provided by a recently found conserved spacer element in σ^E^ promoters [20], which we will analyze in the next subsection.

### Correlations with the conserved spacer element strength

To investigate if the conserved spacer element in σ^E^ is involved in mix-and-matching, we explore to what extent it complements the strengths of the other promoter elements. To that end, we perform an equivalent correlation analysis, as done for canonical σ^E^ promoter elements, which can also provide information about the role of the spacer motif in σ^E^ promoter functioning. In the correlation analysis we include the previously defined σ^E^ promoter datasets with the promoters that are *inactive* in-vitro, all promoters, and promoters *active* in-vitro. Besides correlating the spacer element with the remaining element/parameter strengths, we also re-estimate the previously obtained correlations (between the canonical promoter elements), but now with the newly introduced spacer element strength.

**Fig. 5:**
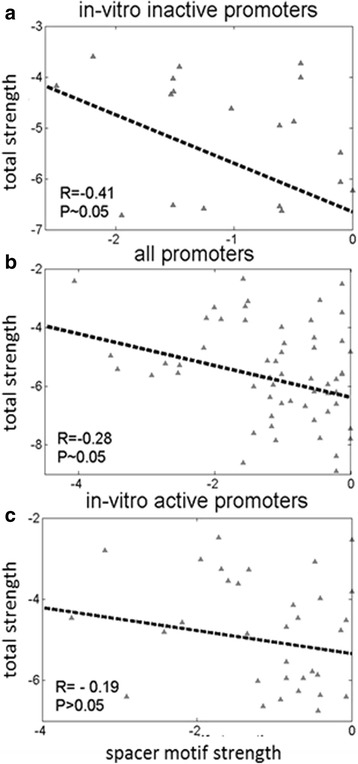
Correlations between the spacer element and the total promoter strength: The upper (**a**), middle (**b**) and lower (**c**) panels in the figure correspond, respectively, to in-vitro *inactive*, all promoters and in-vitro *active* sets. In all three panels the spacer motif strength is correlated with the total promoter strength. The correlation constants and *P* values are also indicated for each panel. The total promoter strength corresponds to the sum of all the element strengths (the strength of the spacer element is not included in the sum)

In the in-vitro *inactive* promoter set, the spacer element makes notable negative correlations with all the promoter elements (ranging from −0.24 to −0.41) (results not shown); the only exception is the positive correlation between the spacer element and the extended −10 element, which may indicate that they jointly complement the strengths of the remaining promoter elements. The largest negative correlation (−0.41), is obtained with the total promoter strength (Fig. 5a), while the lowest correlation is obtained with dsDNA binding elements (−0.24). These notable negative correlations are in line with the assumption that the conserved spacer element mix-and-matches with the canonical promoter elements to achieve a sufficient value of the relevant kinetic parameters.

**Fig. 6:**
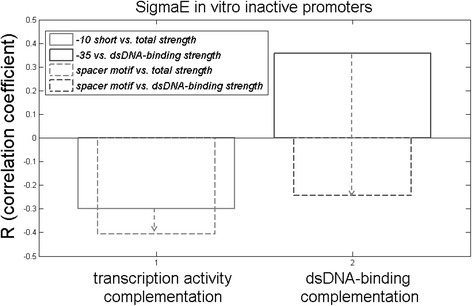
Spacer element vs. canonical elements correlations in the in-vitro *inactive* σ^E^ promoters: The complementation (towards the transcription activity – in the left; towards the dsDNA-binding affinity – in the right) is indicated for the canonical promoter elements, and for the conserved spacer element; the magnitude of the relative R change is indicated by the *dashed arrows*, while the absolute correlation coefficients are provided as values indicated on the *y*-axis; the information on the specific promoter elements involved in the correlations shown is provided in the figure legend

Therefore, we further investigate towards what kinetic parameter is the spacer motif predominantly mix-and-matched with the other promoter elements. To that end, we investigate how including the spacer motif together with the canonical promoter elements changes the negative correlations in σ^E^ in-vitro *inactive* promoters. In Fig. 6, we see that including the spacer motif leads to a large increase in the negative correlations for dsDNA binding complementation (a change of −0.6), and a notably smaller increase in the total transcription activity complementation (a change of −0.1). This clearly indicates that the spacer element has the proposed external factor role in complementing the weak promoters for, their otherwise low, dsDNA-binding affinity. Hence, the largest absolute correlation that is obtained for the spacer element with the total promoter strength is actually a consequence of notable negative correlations that this element accomplishes with almost all the other promoter elements, including those involved in dsDNA interactions. Finally, we also re-estimated the negative correlations between canonical promoter elements and the total transcription activity, once the spacer motif is also included. A consistent increase in the negative correlations is obtained upon this inclusion – for example, the correlation between dsDNA binding elements and short −10 element increases from −0.3 to −0.42. Such increase is also consistent with the spacer motif being involved in complementing the strength of the other promoter elements. The only exception is the extended −10 element, whose correlations with the other promoter elements decrease upon including the conserved spacer motif. This is again consistent with the notion that the spacer motif and the extended −10 element work together in mix-and-matching with the other promoter elements.

Further, we investigate the correlations related with the spacer motif in the *all* promoters set. We still observe negative correlations, but they now decrease with respect to those found in the in-vitro *inactive* set. In particular, the correlations with total promoter strength decrease from −0.41 to −0.28 (Fig. 5b), though this smaller correlation is still statistically significant. Similarly to the results obtained for the in-vitro *inactive* promoters, the negative correlations between the other promoter elements also increase in the set of all promoters, once the spacer element is included.

Finally, we assess the correlations in the in-vitro *active* promoter set, where all the correlations now decrease with respect to the in-vitro *inactive* and the all promoter set, and become statistically insignificant. Particularly, in Fig. 5c, we observe that the correlation between the spacer element strength and the total promoter strength is statistically insignificant and equals −0.19. This result also provides a clear explanation for the previously observed decrease in the respective correlations (Fig. 5, a and b), between the in-vitro *inactive* and all promoters sets, which is due to including the in-vitro *active* sequences in the all promoter set. Note that the observed decrease of correlations from the in-vitro *inactive* to in-vitro *active* sequences – i.e. a smaller functional significance of the motif in the in-vitro *active* sequences – is consistent with a less pronounced presence of the spacer motif in the in-vitro *active* compared to the in-vitro *inactive* set [20]. Consequently, the main function of the spacer element is to complement dsDNA binding strength in otherwise weak in-vitro *inactive* promoters.

## Discussion

The main hypothesis in this work is that the mix-and-matching mechanism, which has been well established in Group I (RpoD) σ factors, is also present for ECF σ family members. To investigate this hypothesis, we here examined if the strengths of the promoter elements for the canonical ECF σ members exhibit mix-and-matching, as this would imply ubiquity of the mechanism in the entire family, since ECFs are the most divergent σ^70^ factors with respect to RpoDs. We also compared the observed complementation with an equivalent correlation analysis in RpoDs, which allowed investigating what are the relevant kinetic parameters that define a functional promoter within ECF σ and RpoD groups. The obtained results are further discussed below.

**Fig. 7:**
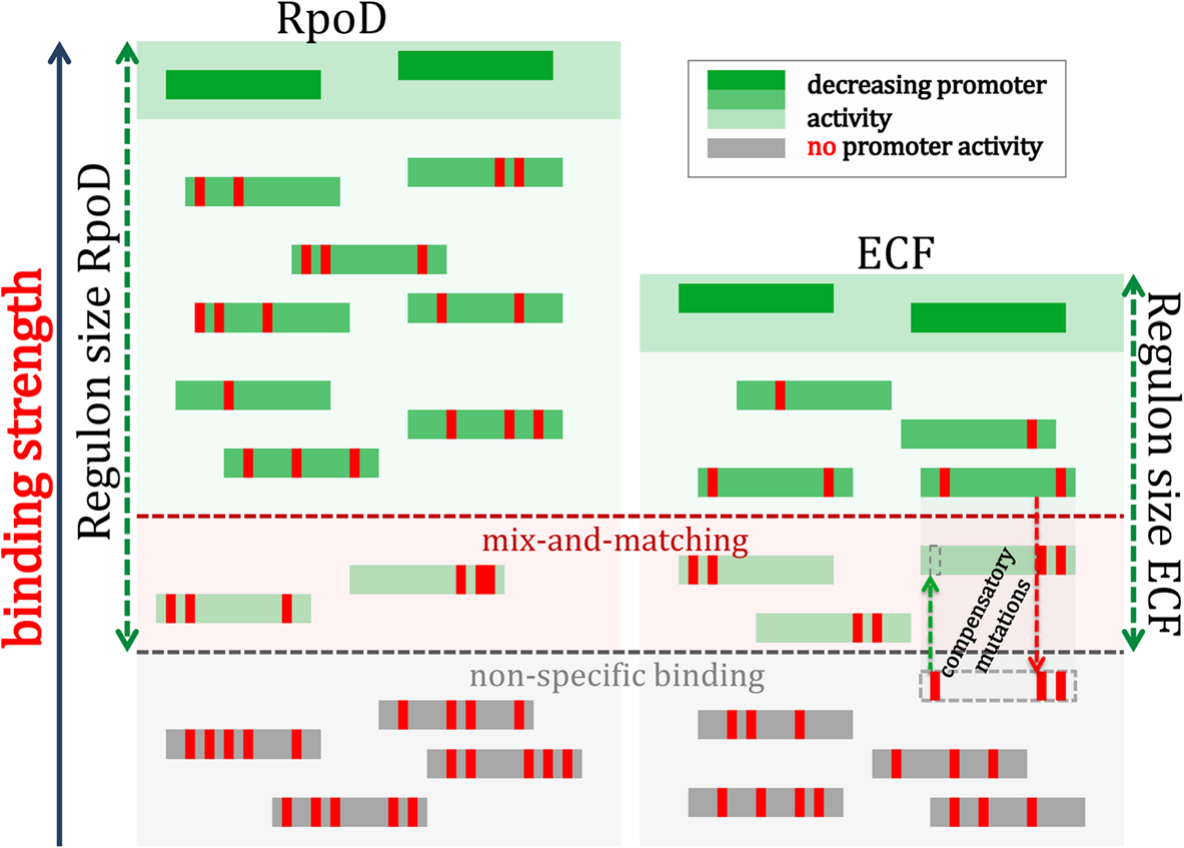
Relation of the mix-and-matching with σ factor specificity: A regulon of a σ factor with low specificity (RpoD - on the left side) and high specificity (ECF - on the right side) is shown in the figure; promoter sequences are indicated by the *green rectangles* (where the strongest shade of green corresponds to the consensus promoter sequences); mutations in the promoters are indicated by the *vertical red rectangles*; the zone of mix-and-matching, where the sequences maintain their promoter activity by introducing compensatory mutations, is *shaded red*; the zone of non-specific binding, where the sequences have lost their promoter activity, is *shaded grey*

In general, though the obtained correlations are statistically significant, a notable scatter can be also observed in the plots. As an example, the largest correlation obtained in RpoD group, where the mix-and-matching mechanism has been well established, is around −0.2. This might appear counter-intuitive, since RpoD σ factors are characterized by a large sigmulon size, which is naturally associated with lower conservation of the implicated promoter elements (i.e. lower specificity) thus seemingly providing a higher probability for mix-and-matching between the elements to arise. To understand this seemingly unintuitive result, we consider a model which is summarized in Fig. 7. The model is based on an assumption that for a majority of the promoter sequences, other constraints arise, such as tuning a desired level of the promoter activity or binding strength to dsDNA. Therefore, only a small fraction of the promoter sequences, which are close to the threshold that distinguishes specific from non-specific binding, have to resort to mix-and-matching for maintaining sufficiently high level of the relevant kinetic parameter (e.g. the transcription activity for RpoDs). In particular, in Fig. 7 we divide the space of the promoter sequences in three regions: i) The region of higher promoter activity (the uppermost region in the figure), where the promoter strength is away from the non-specific boundary, and where mutations accumulate to tune the transcription activity to a desired level. Note that in the figure we indicate a decrease in the promoter strength, as mutations are introduced in the promoter elements with respect to the consensus sequence. ii) The region of mix-and-matching, where the transcription activity comes close to the non-specific boundary. In this region, there is a strong selection pressure on the promoters to remain functional (under the assumption that the environment is such that the unbroken transcription link confers positive selection), which is exhibited through mix-and-matching of the promoter element strengths. In particular, a mutation that would decrease strength of one promoter element can be met by a compensatory mutation that would increase strength of the other promoter element (indicated in the figure), so that a minimal value of the relevant kinetic parameter is preserved. iii) The region of non-specific binding, where a sufficiently large number of mutations makes the promoter non-functional. In accordance with this model, the larger sigmulon size does not imply larger negative correlations between the promoter elements. On the contrary, the smaller sigmulon size (as e.g. indicated for ECF σ in the figure) would imply a larger fraction of the total number of promoters in the region of mix-and-matching – i.e. in such case there is a narrower range for the promoters to accumulate mutations, without them falling near the non-specific binding boundary (in the zone of mix-and-match). Consequently, larger negative correlations would be expected in this case.

The results that we obtained are in accordance with this model, i.e. the negative correlations are indeed more pronounced in ECF promoter sequences (σ^E^ in *E. coli*). For example, for σ^E^ promoters *active* in-vitro, the mutual complementation of dsDNA binding elements is significantly stronger than in RpoD promoters (more than −0.4 vs. -0.1). This result becomes also important from another perspective, since the complementation of dsDNA binding elements has been originally proposed as the mix-and-matching mechanism in RpoDs [5], but is now actually observed for ECF σ factors. Therefore, it is the binding affinity to dsDNA what distinguishes a functional promoter for ECF σs, i.e. there is clearly a selection pressure on the promoter elements that are involved in dsDNA interactions, to complement each other strengths. This appears plausible from the point of ECF σ physiological response, as it may provide a more efficient recruitment of ECF σ to its promoters, which is, in turn, likely important for the highly focused and rapid response to the outside stimulus, that is expected from these σs.

Conversely, in the RpoD group, it is the total transcription activity, rather than the binding affinity, which is associated with complementation of the promoter element strengths. This also appears plausible, as the housekeeping σ factors are likely not under a constraint for fast responsiveness to external stimulus, that would have to be met by a sufficiently strong binding affinity to dsDNA. Consequently, while mix-and-matching emerges as a general mechanism of promoter recognition in the σ^70^ family, there are likely significant differences in the relevant kinetic parameters, that may ensure accounting for diversity of physiological responses that need to be exhibited by the different groups of σ factors.

In line with this, it becomes clear that mix-and-matching may be mechanistically exhibited by different combinations of promoter elements in different groups of σ factors. In particular, in RpoD σs the greatest extent of mix-and-matching is observed between −15 element and the remaining elements, while the mutual complementation between −35 and −10 elements is not significant. Such a distinguished role of −15 element in RpoDs may be a direct consequence of its interactions with a separate domain of σ factor (domain σ_3_ of RpoDs). On the other hand, in ECF σs, all the promoter elements mix-and-match on about an equal scale, which may be related with a much simpler protein structure in this group, with only two distinguished DNA-recognition domains (σ_2_ and σ_4_).

Moreover, one can notice that the correlations between dsDNA elements significantly decrease when moving from the in-vitro *active* to in-vitro *inactive* promoter set in ECF σs. This decrease is likely due to the fact that weak promoters depend on external factors for their activity, which spoils mix-and-matching between the canonical promoter elements. Interestingly, negative correlations between ssDNA and dsDNA promoter elements remain significant even for the in-vitro *inactive* sequences. This is likely due to the low-activity promoters experiencing a large pressure to sufficiently increase their inherently low transcription activity. The similar trend of promoter element complementation (mix-and-matching), between ssDNA and dsDNA binding elements, was also observed for σ^W^.

The proposal that the external factors might be involved in mix-and-matching, with the role of helping weak promoters to accomplish a sufficient level of the relevant kinetic parameter for transcription initiation, is further supported by our analysis of the complementation associated with the conserved σ^E^ spacer element. Here, the strongest negative correlations are in the in-vitro *inactive* set (where the spacer element is much more pronounced). Moreover, the complementation towards dsDNA-binding affinity experiences the largest relative increase when the spacer motif is taken into account, compared to the correlations obtained for the canonical promoter elements. Finally, there is a clear increase in the negative correlations between the other promoter elements in the in-vitro *inactive* set, upon including the spacer motif. This then clearly identifies the spacer element as one major additional factor, on which depends the initiation from weak σ^E^ promoters. Finally, the role of the spacer element as an external contribution to the weak promoter activity (i.e. dsDNA-binding affinity) is further established by an almost complete absence of both the spacer element, and the correlations associated with it, from the in-vitro *active* set – which is otherwise characterized by the strongest negative correlations among the canonical promoter elements, especially the ones involved in dsDNA interactions.

Furthermore, transcription initiation implies concert participation of the whole body of RNA polymerase holoenzyme in the interaction with promoter DNA. Consequently, other examples of the external promoter signatures that are involved in mix-and-matching may be provided by the interactions of core RNA polymerase with promoter, such as the interactions of the αCTDs with the UP elements [18], or of the β and β’ subunits with the downstream duplex promoter segments [14, 16]. Moreover, involvement of core RNAP subunits may bring invariant impact in transcription initiation, e.g. intramolecular rearrangements involving β and β’ subunits can in a similar way stabilize the open promoter complex formation with different σ subunits [17], therefore also contributing to the universal character of mix-and-matching. Finally, the external promoter signatures, which are involved in mix-and-matching, may be also provided by transcription factor binding sites that regulate expression of ECF σ promoters [25, 26], though such regulation appears to be understudied.

We here presented a detailed analysis of the natural set of promoter sequences, where through the correlation analysis we detect selection pressures that act on these sequences, i.e. which force them to complement the strengths of the relevant promoter elements to remain functional. Another approach to investigating mix-and-matching would be biochemical, i.e. can be exhibited through in-vitro transcription analysis, where one can start from a specific promoter, and mutate its promoter elements (e.g. by changing one bp. at a time), while observing if compensatory mutations preserve the promoter functionality. Such analysis has been previously performed for RpoDs, but to our best knowledge, not for the alternative σ factors. In fact, the study presented here would be largely complementary to such biochemical analysis – i.e. while we analyzed the selection pressures acting on the promoter elements, the biochemical analysis would assess mechanistic constraints imposed in such mix-and-matching. More widely, a better characterization of both the promoter sequences (along the lines done for σ^E^), and in-vitro biochemical measurements of the mutated sequences, might establish mix-and-matching as a common promoter recognition mechanism in the entire σ^70^ family.

## Conclusion

In contrast to the previous assumptions, we have found that the mix-and-matching mechanism is also exhibited in ECF σ^70^ subfamily (σ^E^ in *E. coli*), with even stronger correlations than those observed in RpoD group. We have also distinguished the relevant kinetic parameters of promoter recognition for different σ^70^ groups – i.e. dsDNA-binding affinity for ECFs and total transcription activity for RpoDs – which are accomplished mechanistically through different combinations of promoter elements involved in mix-and-matching. Additionally, we have also shown that, in weak promoters, external factors, such as the newly found conserved spacer element in σ^E^ promoters, mix-and-match with canonical promoter elements for achieving a sufficient level of the relevant kinetic parameter (e.g. dsDNA-binding affinity for the in-vitro *inactive* σ^E^ promoters). We also proposed a simple model, which relates the extent of mix-and-match and the σ factor specificity (i.e. the sigmulon size). The model is based on the assumption that it is mostly the promoter sequences in the relative vicinity of the non-specific binding threshold that are under the selection pressure to exhibit mix-and-matching. Contrary to intuitive expectations, but consistent with our results, the model predicts that smaller regulon size is related with the larger extent of mix-and-matching. Overall, the evidence of mix-and-matching in ECF σ subfamily, which is the most distant from RpoDs, suggests that mix-and-matching may be a common promoter recognition mechanism in the entire σ^70^ family, which should be tested by future more detailed analysis of the entire σ^70^ family. Such finding would be highly significant, as it may provide a unifying framework for understanding promoter recognition within the diverse σ^70^ family.

## Methods

We here summarize a biophysical model of transcription initiation [13, 27], and provide a relationship between the weight matrix scores and kinetic parameters of transcription initiation.

### Biophysical model of transcription initiation

#### The kinetic scheme and parameters

We start with the biophysical model of transcription initiation, previously developed in [13], that is based on the following general scheme of transcription initiation [10]:

1.1 $$ \left[ RNAP\right]+\left[P\right]\underset{k_{off}}{\overset{k_{on}}{\rightleftarrows }}{\left[ RNAP-P\right]}_c\overset{k_f}{\to }{\left[ RNAP-P\right]}_o\overset{k_e}{\to }{\left[ RNAP\right]}_e+\left[P\right] $$

where RNA polymerase, promoter DNA, closed and open RNAP-promoter complexes, are denoted as [*RNAP*], [*P*], [*RNAP-P*]_*c*_ and [*RNAP-P*]_*o*_, respectively. The on and off rates of RNAP binding to the promoter are denoted as *k*_*on*_ and *k*_*off*_, the transition rate from closed to open complex as *k*_*f*_, while the rate of RNAP escape from the promoter as *k*_*e*_. Thus, the first step in the scheme denotes reversible binding of RNAP to the promoter, which is followed by the opening of the two DNA strands and forming the open complex, as illustrated by the second step in the scheme. The last step is the irreversible RNAP promoter escape, followed by RNAP transition to the elongation.

As RNAP binds and unbinds on a much faster timescale (~1 s) compared to subsequent transition from closed to open complex (~100 s) [10], and since the promoter is occupied by RNAP only a fraction of the time (the “unsaturated approximation”) [23], the expression for transcription activity can be simplified to [13]:

1.2 $$ \varphi \approx \left[ RNAP\right]\;{K}_B\kern0.1em {k}_f, $$

where [*RNAP*] denotes the concentration of free RNAP in the cell. Note here that transcription activity, is in Eq. (1.2) directly proportional to *K*_*B*_ *k*_*f*_, whereby this product (of the binding affinity and the transition rate) corresponds to the usual measure of the promoter strength [10].

#### The relation with the interaction energies

The kinetic parameters are directly related to the interaction energies between σ factor and the promoter DNA. First, we start with the binding affinity of RNAP to dsDNA, which depends on the interaction energies of σ factor with −35 element, with dsDNA segment of −10 element, and with the length of the spacer sequence between −35 and −10 elements [12, 13]:

1.3 $$ \log \left({K}_B(S)\right)\sim c-\frac{\varDelta {G}_{ds}\left({S}_{\left(-35\right)}\right)+\varDelta G\left(\gamma \right)+\varDelta {G}_{ds}\left({S}_{\left(-10\right)}^{(ds)}\right)}{k_B\kern0.1em T}, $$

where *S*_(−35)_, *S*_(−10)_^(*ds*)^ and *γ* denote, respectively, sequences of −35 element, the dsDNA −10 element segment, and the spacer length, while *c* is a sequence-independent constant. *ΔG*_*ds*_(*S*_(−35)_), *ΔG*_*ds*_(*S*_(−10)_^(*ds*)^) and *ΔG*(*γ*) are, respectively, the interaction energies of σ factor with −35 element, dsDNA segment of −10 element, and the differences of the interaction energies due to the variable spacer length.

Moreover, for relating *k*_*f*_ with the interaction energies, we use the mechanistic model of the open complex formation [13]:

1.4 $$ \log \left({k}_f\right)=c+\frac{\varDelta {G}_m\left({S}_{\left(-10\right)}^{(ss)}\right)-\varDelta {G}_{ss}\left({S}_{\left(-10\right)}^{(ss)}\right)}{k_B\kern0.1em T} $$

where *S*_(−10)_^(*ss*)^ denotes the −10 element segment which is melted during the open complex formation (interacts with the σ factor in ssDNA form). *ΔG*_*m*_(*S*_(−10)_^(*ss*)^) denotes the energy of opening *S*_(−10)_^(*ss*)^ in the absence of RNAP (the DNA melting energy), while *ΔG*_*ss*_(*S*_(−10)_^(*ss*)^) denotes the interaction energy of σ factor with ssDNA sequence *S*_(−10)_^(*ss*)^ in the open complex.

From the expressions given above, the transcription activity of the promoter sequence *S* can be expressed in terms of the interaction energies (all the terms are defined above):

1.5 $$ \log \left(\varphi (S)\right)=c+\frac{-\varDelta {G}_{ds}\left({S}_{\left(-35\right)}\right)-\varDelta G\left(\gamma \right)-\varDelta {G}_{ds}\left({S}_{\left(-10\right)}^{(ds)}\right)+\varDelta {G}_m\left({S}_{\left(-10\right)}^{(ss)}\right)-\varDelta {G}_{ss}\left({S}_{\left(-10\right)}^{(ss)}\right)}{k_B\kern0.1em T}. $$

#### Parameterizing the model by the weight matrices

To parameterize Eqs. (1.3)-(1.5), we use the independent nucleotide approximation [28–30], where the interaction energies are provided by the sum of the terms corresponding to different bases at different positions in the DNA motifs involved in the σ factor binding. It has also been shown previously that the protein-DNA interaction energies for a given base at a given position in the motif correspond to the weight matrix elements [22, 23], so that *K*_*B*_(*S*) and *φ*(*S*) can be expressed as follows:

1.6 $$ \log \left({K}_B(S)\right)\sim {\displaystyle \sum_{i=-35}^{-30}{\displaystyle \sum_{\alpha =1}^4{w}_{i\alpha}^{\left(-35\right)}\kern0.1em {S}_{i\alpha}^{\left(-35\right)}}}+{\displaystyle \sum_{j=1}^5{w}_j^{\left(\gamma \right)}{\delta}_{j\gamma }}+{\displaystyle \sum_{i=-15}^{-12}{\displaystyle \sum_{\alpha =1}^4{w}_{i\alpha}^{\left(-10\right)}\kern0.1em {S}_{i\alpha}^{\left(-10\right)}}} $$

1.7 $$ \log \left(\varphi (S)\right)\sim {\displaystyle \sum_{i=-35}^{-30}{\displaystyle \sum_{\alpha =1}^4{w}_{i\alpha}^{\left(-35\right)}\kern0.1em {S}_{i\alpha}^{\left(-35\right)}}}+{\displaystyle \sum_{j=1}^5{w}_j^{\left(\gamma \right)}{\delta}_{j\gamma }}+{\displaystyle \sum_{i=-15}^{-12}{\displaystyle \sum_{\alpha =1}^4{w}_{i\alpha}^{\left(-10\right)}\kern0.1em {S}_{i\alpha}^{\left(-10\right)}}}+{\displaystyle \sum_{i=-11}^{-7}{\displaystyle \sum_{\alpha =1}^4{w}_{i\alpha}^{\left(-10\right)}\kern0.1em {S}_{i\alpha}^{\left(-10\right)}}} $$

where *w*_*iα*_ denotes the weight matrices, and the superscripts ((−35), (−10) or (*γ*)) indicate that these matrices correspond, respectively, to −35 element, −10 element or the spacer length. Different positions within −35 and −10 promoter elements are marked with the index *i*, whereas the index *j* denotes five possible spacer lengths.

In summary, from Eq. (1.6), we see that the weight matrix scores of the promoter elements that interact with σ factor in dsDNA form contribute additively to the log binding affinity - log(*K*_*B*_(*S*) ). Similarly, from Eq. (1.7), we see that the log transcription activity log(*φ*(*S*) ), is obtained by summing the weight matrix scores of the promoter elements which interact with σ factor in ssDNA form. These relations between the weight matrix scores and the relevant kinetic parameters were used for analyzing how the promoter elements complement each other strengths.

### DNA sequences

σ^E^ promoter dataset is composed of 60 experimentally verified promoters with aligned −35 and −10 elements, divided according to the level of transcription activity under the in-vitro conditions [21]. σ^W^ promoter dataset was composed of 34 experimentally determined promoters, with one promoter sequence (upstream of ywbLMN) omitted, due to the difficulty in aligning its −35 element (bearing at least five mismatches from the consensus). These promoters were retrieved from the DBTBS database, that contains information on *Bacillus subtilis* promoters and σ factors [24]. RpoD promoter dataset is composed of 322 sequences with experimentally determined transcription start sites, retrieved from the RegulonDB database.

All the sequences were *de novo* aligned through Gibbs Motif Sampler, in the Motif Sampler mode, by searching only the direct strand, setting the number of motifs to 1 and the total number of sites to the number of query sequences. The motif length was set to several different values for each promoter set (to verify the robustness of the detected motif), while the remaining parameters were at their default values.

### Constructing weight matrices

The weight matrix elements- *w*_*α,i*_, defining weights for base *α* at position *i* in the motif are calculated as [28, 31]:

$$ {w}_{\alpha, i} = \log \left(\frac{n{v}_{a,i} + {p}_a}{p_a\left(n+1\right)}\right) $$

where *n* is the number of motifs in the alignment, *v*_*a,i*_ is the frequency with which the base *α* appears in the alignment at the position *i,* and *p*_*a*_ represents the base background frequency; adding of *p*_*a*_ in the numerator corresponds to the pseudocounts.

Weights, corresponding to different spacer lengths, are calculated according to [7]:

$$ {w}_i = \log \left({v}_i\right) $$

where *w*_*i*_ represents the weight of a spacer of length *i*, and *v*_*i*_ is the spacer frequency.

Also, from each column of a weight matrix, we subtracted the value that corresponds to the consensus base, so that the score of the consensus motif becomes zero.

Specifically, weight matrices were constructed to assess correlations of the motif strengths in σ^E^ and σ^W^ promoters (datasets described above) – for σ^E^ we used the alignments of its −35 elements, extended −10 elements, short −10 elements, and the alignment of the spacer motif identified in this study (for reference on promoter elements definition see Fig. 1 in Introduction); for σ^W^ we used the −35 elements alignment.

### Correlating motif strengths

Correlation constants were determined by using a MATLAB (Mathworks) routine. The same MATLAB function allows calculating *P* values of the obtained correlation constants. Briefly, the routine is based on randomly permuting the points in the dataset; correlation constant for each random permutation is calculated, and statistical significance of the difference between the original correlation constant and the correlation constants in the permuted dataset is estimated by using t-test.

## Additional file

Additional file 1: Mix-and-matching in σ^W^ sequences. (PDF 79 kb)
