# Supplementary material for: Mix-and-matching as a promoter recognition mechanism by ECF σ factors
Source: BMC Evol Biol. 2017 Feb 7;17(Suppl 1):12. doi: 10.1186/s12862-016-0865-z (PMC5333181; doi:10.1186/s12862-016-0865-z)
Supplement: Additional file 1: — Mix-and-matching in σW sequences. (PDF 79 kb) [file 12862_2016_865_MOESM1_ESM.pdf]

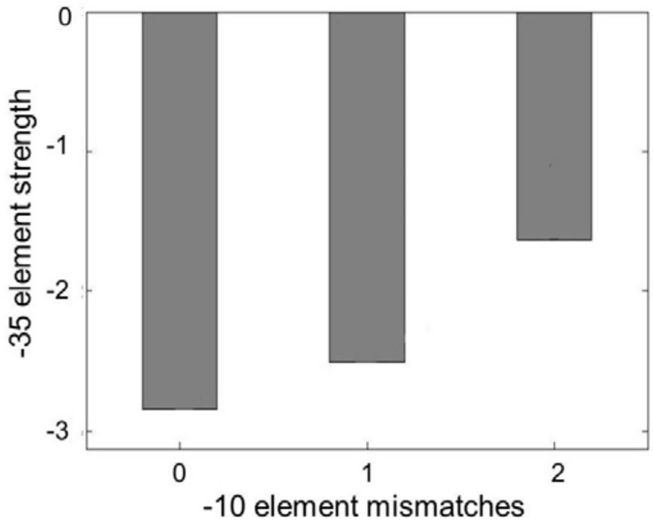

Supplement Figure 1: Mix-and-matching in σ^W sequences. A correlation between the average dsDNA element strength (assessed by the weight matrix score – *y*-axis) and ssDNA element strength (assessed by the number of mismatches in the corresponding -10 elements – *x*-axis) is shown. Note that stronger -35 element strength is associated with larger (less negative) weight matrix score.
